# First person – Mohd. Salman

**DOI:** 10.1242/dmm.046169

**Published:** 2020-08-19

## Abstract

First Person is a series of interviews with the first authors of a selection of papers published in Disease Models & Mechanisms, helping early-career researchers promote themselves alongside their papers. Mohd. Salman is first author on ‘Nrf2/HO-1 mediates the neuroprotective effects of pramipexole by attenuating oxidative damage and mitochondrial perturbation after traumatic brain injury in rats’, published in DMM. Mohd. is a PhD student in the lab of Prof. Suhel Parvez at Jamia Hamdard, New Delhi, India, investigating the cellular and molecular mechanisms involved in traumatic brain injury, ischemic stroke and neurodegenerative diseases, and exploring the avenue of drug repurposing with pre-clinical studies.


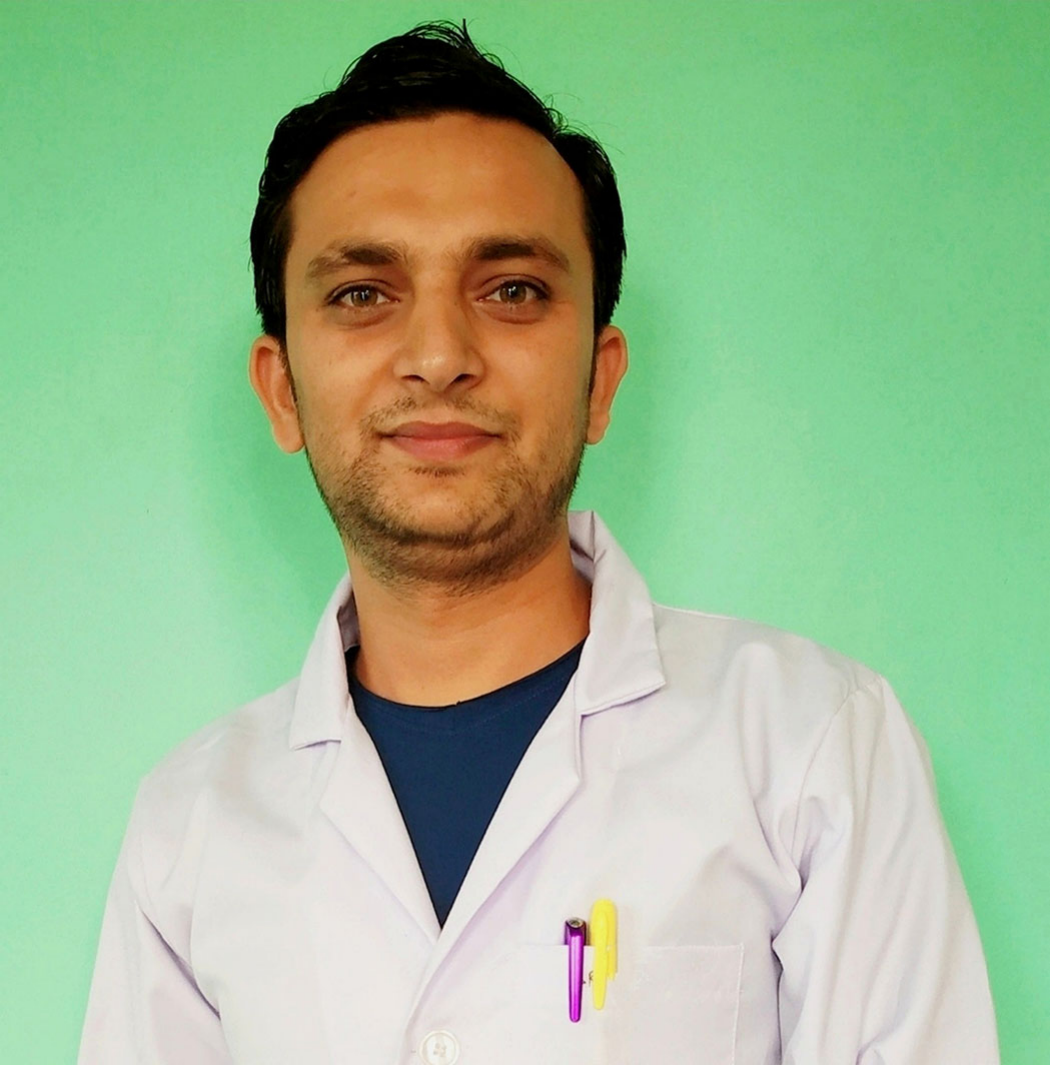


**Mohd. Salman**

**How would you explain the main findings of your paper to non-scientific family and friends?**

Usually, I need to simplify my research work quite a lot when I am explaining it to my family and friends. I start by telling them that I am studying the role of mitochondria in traumatic brain injury (TBI) and our goal is to find a new neurotherapeutic agent so that we can prevent the worst outcomes after brain injury. In simple words, TBI is a severe threat to human health among the several existing neurological disorders. It is associated with difficulties in communications and behavioral impairments, which in turn contribute to a socioeconomic burden for people of all ages throughout the world. Currently, there are no treatments that can reduce or prevent brain injury. In this particular study, we used a controlled cortical impact injury model of TBI in Wistar rats to investigate the therapeutic effects of a dopamine receptor agonist (pramipexole) for drug repurposing purposes. The concept of drug repurposing is utilizing an existing therapeutic to treat a new disease, and holds the promise of fast clinical impact at lower cost than *de novo* drug development and known safety profiles. We found that pramipexole significantly attenuates neurobehavioral alterations, oxidative damage and mitochondrial perturbation after TBI via regulating the Nrf2/HO-1 signaling pathway. Therefore, our research findings support that pramipexole is a potential therapeutic candidate against brain injury.

“[…] neurotrauma is still a step ahead of researchers […]”

**What are the potential implications of these results for your field of research?**

Even though neurotrauma is a heterogeneous neurological disorder and has been studied extensively, we have limited means to prevent injury after TBI. It seems that neurotrauma is still a step ahead of researchers, and we have a lot to investigate to overcome this challenge (among oxidative damage, mitochondrial dysfunction, neurodegeneration and neuroprotection) in rodent models. Our present study demonstrates that pramipexole provides neuroprotective effects by activation of the Nrf2/HO-1 pathway following TBI. Importantly, we hope this research work provides a convincing example of drug repurposing. It would be great to see more researcher groups choosing drug repurposing in other disease models for their pre-clinical studies. This could have a positive impact on the whole drug repurposing concept.

**What are the main advantages and drawbacks of the model system you have used as it relates to the disease you are investigating?**

In the present study, we have used a chronic constriction injury model of TBI in Wistar rats. This model allows for quantitative control over traumatic injury force, velocity, depth and dwell time, as well as extent of the brain tissue deformation. Thus, it produces control over biochemical parameters and functional alterations known to be associated with the brain injuries. This independent control over the brain injury parameters across a broad range of contact velocities contributes to the consistency and accuracy of controlled cortical impact as a model of TBI in rodents. This model is clinically relevant to mimic features of accidental brain injury in humans and widely used for pre-clinical studies for TBI. In addition, we used an animal model to study TBI pathogenesis and this model can fill the needs for a test bed for novel drugs and treatments in pre-clinical studies. There are, however, some limitations as this model cannot completely replicate the symptom profiles experienced by humans, which is definitely a drawback.

**What has surprised you the most while conducting your research?**

While conducting the research experiments, I was very impressed by the chronic constriction injury model and its reliability of inducing brain injury in the experimental rodents. The extent of brain injury was reproducible, to a large extent, and was significant. Observing the mitochondrial dysfunction and protective effects of pramipexole through the Nrf2/HO-1 signaling pathway, which we evaluated with different parameters, was a fascinating and beautiful journey for me.

**Describe what you think is the most significant challenge impacting your research at this time and how will this be addressed over the next 10 years?**

Currently, the major challenge is deciphering the path to prevent the worst side effects of neurotrauma in humans. The uncertainty and unpredictability in what kind of impact of injury can induce brain trauma is a major challenge. A more comprehensive understanding of mitochondrial perturbation following TBI along with the role of mitochondrial dynamics needs to be explored. Our results convincingly show the neuroprotective effects of dopamine D2 agonists like pramipexole, and the signaling pathway associated with different outcomes of brain injury, in a rodent model. I think that in the next decade these data can be better utilized to form a more uniform picture of mitochondrial-mediated neuroprotection against brain injury and other neurological disorders. This should unequivocally support the drug repurposing concept and the use of different receptor agonists/antagonists, and testing them in other neurological diseases with pre-clinical studies.

**Figure:**
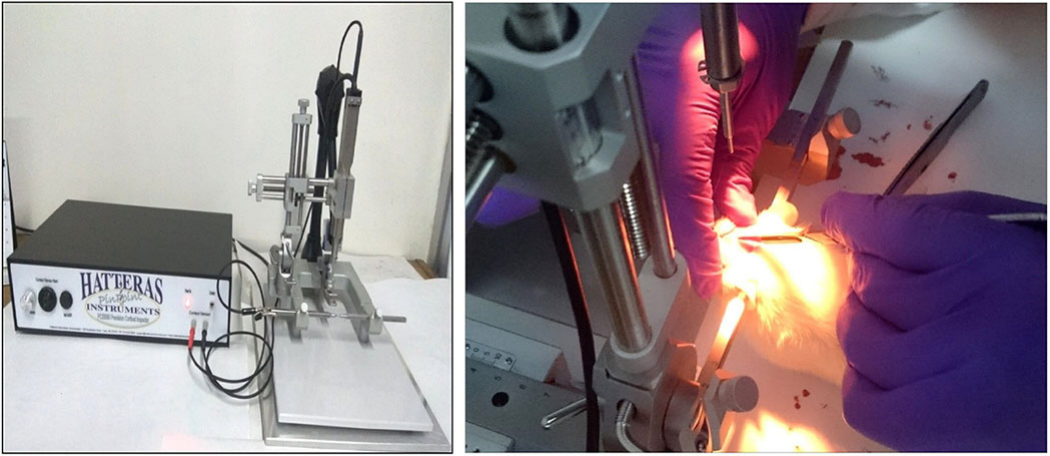
**Controlled cortical impact model of traumatic brain injury.**

**What changes do you think could improve the professional lives of early-career scientists?**

There are many things that are well organized in our university. My department, in particular, takes adequate care in fulfilling the requirements of doctoral students and postdoctoral fellows. We have lots of peer support, colloquiums, student research advisory committees, lectures on research methodology and follow-up groups. There are extramural funding agencies at a national level, which makes it possible for us to take part in national and international conferences in the neuroscience field. There are, however, a few things that can be improved for early-career scientists from an Indian perspective. The one thing that is lacking currently is to have some kind of mentoring program, so that mentors could give support throughout the project, and encourage setting further goals and thinking about your career in a broader sense. This could increase self-confidence and motivation in early-career scientists. Additionally, these mentors have to be from the leading research groups worldwide, who – in this digital era – can help us to bridge the gap.

**What's next for you?**

My research work so far has been interesting and I would like to study mitochondrial dynamics in neurological/neurodegenerative diseases. For me, the next goal is to complete my doctorate degree within the next couple of months and to find a challenging interesting postdoctoral position in neuroscience.
